# Cost-effective, user-friendly detection and preconcentration of thrombin on a sustainable paper-based electrochemical platform

**DOI:** 10.1007/s00216-025-05764-9

**Published:** 2025-02-01

**Authors:** Ada Raucci, Giuseppina Sorrentino, Sima Singh, Nicola Borbone, Giorgia Oliviero, Gennaro Piccialli, Monica Terracciano, Stefano Cinti

**Affiliations:** 1 Department of Pharmacy, University of Naples “Federico II, ” Via Domenico Montesano 49, 80131 Naples, Italy; 2 Department of Molecular Medicine and Medical Biotechnologies, University of Naples Federico II, via Sergio Pansini 5, 80131 Naples, Italy; 3 Sbarro Institute for Cancer Research and Molecular Medicine, Center for Biotechnology, College of Science and Technology, Temple University, Philadelphia, PA 19122 USA; 4 Bioelectronics Task Force at University of Naples Federico II, Via Cinthia 21, 80126 Naples, Italy

**Keywords:** Thrombin, Paper-based, Screen-printed electrodes, Point of care, Aptasensor

## Abstract

Thrombin overexpression in serum serves as a critical biomarker and is implicated in several diseases associated with significant morbidity and mortality. Existing techniques for thrombin detection are time-consuming and require sophisticated equipment and extensive sample preparation procedures, which further delay the detection and increase the cost of the procedure. Early and accessible diagnosis at the point of care, especially in limited-resource countries, represents the first step of clinical interventions. To overcome these limitations, we have proposed an innovative, sustainable paper-based electrochemical detection platform for thrombin. In this work, a sustainable paper-based aptasensor was rationally designed, characterized, evaluated against conventional gold standard plastic-based substrates, and applied to human serum, yielding a detection limit of ~ 60 pM. The present method provides an efficient and user-friendly way for the detection of thrombin and potentially leading to better management and treatment outcomes for patients.

**Graphical abstract:**

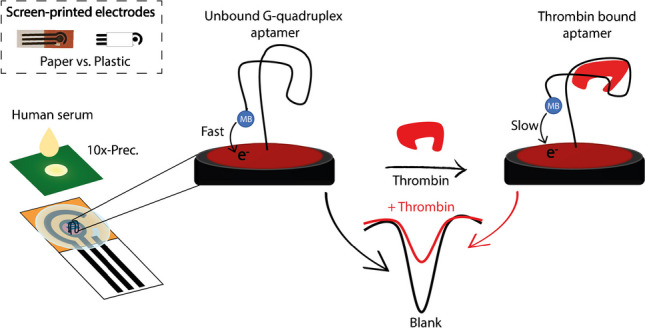

**Supplementary Information:**

The online version contains supplementary material available at 10.1007/s00216-025-05764-9.

## Introduction

Thrombin is an allosteric serine protease that plays a crucial role in the clotting process as part of the coagulation cascade, transforming fibrinogen into fibrin. Beyond coagulation, thrombin regulates different biological processes, acting as both an activator and inhibitor in functions related to normal physiology, diseases progression, and tissue healing along blood vessel walls [1, 2]. The levels of thrombin in blood can vary significantly, ranging from nanomolar [3] to low concentrations, while detection within the picomolar range is vital for specific diagnostic purposes [4]. Its blood occurrence can be related to conditions such as Alzheimer’s disease [5], progression of nephrotic syndrome [6], cardiovascular [7], and pulmonary diseases [8] as well as complications, in tumor advancement and metastasis [9, 10].

Despite its clinical significance, thrombin detection remains challenging, particularly in point-of-care settings. Traditional diagnostic methods are highly sensitive and reliable but require multiple processing steps, costly reagents, and sophisticated laboratory infrastructure, making them time-consuming and inaccessible in resource-limited environments [2, 11, 12]. Thrombin detection in clinical practice employs several classical techniques, each with its specific advantages and limitations. Thrombin generation assays (TGAs), such as the calibrated automated thrombogram (CAT) assay, are valuable in providing a comprehensive view of thrombin production and inactivation, reflecting the balance between procoagulant and anticoagulant activities [13]. However, these tests require specialized equipment, which limits their widespread application in routine clinical settings. Traditional clotting time assays, such as prothrombin time (PT) and activated partial thromboplastin time (aPTT), are widely used but measure only a small portion of thrombin activity and fail to detect hypercoagulability or the contribution of natural anticoagulants, making them less effective for comprehensive thrombin assessment [14, 15]. Immunoassays, including the enzyme-linked immunosorbent assay (ELISA) and western blotting, are sensitive but often require extensive sample processing, which is time-consuming and may not provide real-time results [16, 17]. Similarly, viscoelastic analysis methods, such as thromboelastography (TEG) and rotational thromboelastometry (ROTEM), provide real-time data on clot formation and stability, but do not capture the full dynamics of thrombin generation. Fibrinogen function tests, such as reptilase time, can help assess coagulation status, but they also have specificity limitations [14]. These limitations underscore the challenges associated with traditional diagnostic methods, particularly in point-of-care scenarios where rapid and accurate results are critical. This highlights the growing demand for portable, fast and easy-to-use thrombin detection instruments that achieve levels of sensitivity comparable to standard laboratory tests.

Leveraging this property, numerous portable devices have been successfully designed for thrombin detection [15–19], highlighting the transformative potential of aptamer-based technologies in biosensing applications.

To overcome these challenges, oligonucleotide aptamers have emerged as highly effective recognition elements (i.e., bioprobes) positively impacting the development of sensors and biosensors. The aptamers, selected through a combinatorial procedure known as SELEX [18, 19], are characterized by a high specificity towards a target molecules. They offer several distinct advantages over traditional bioprobes, such as antibodies, due to their unique properties. Unlike antibodies, which require in vivo immunization of animals for production, aptamers can be synthesized using solid-phase oligonucleotide synthesis, ensuring high reproducibility. Additionally, aptamers exhibit greater resistance to heat, pH fluctuations, and organic solvents compared to antibodies or proteins. Their binding affinities and specificities can also be easily optimized and enhanced through appropriate design modifications.

Based on this recognition element, several portable devices have been developed to detect thrombin [20–24]. Among the major features of the aptamer to detect thrombin, they are specificity, quickness of response, and cost-effectiveness [25]. Even if different approaches and transduction methods have been reported, including optical [26], fluorescence [27], electrochemiluminescence (ECL) [28], and surface-enhanced Raman spectroscopy (SERS) [29], electrochemical-based detection still represents a preferred choice to be applied in complex biological matrices without being affected by color/turbidity of solutions [30]. In line with the increasing need for sustainable diagnostic solutions, paper-based devices have emerged as promising alternatives due to their low cost, lightweight nature, and compatibility with real biological samples [27, 28].

**Fig. 1:**
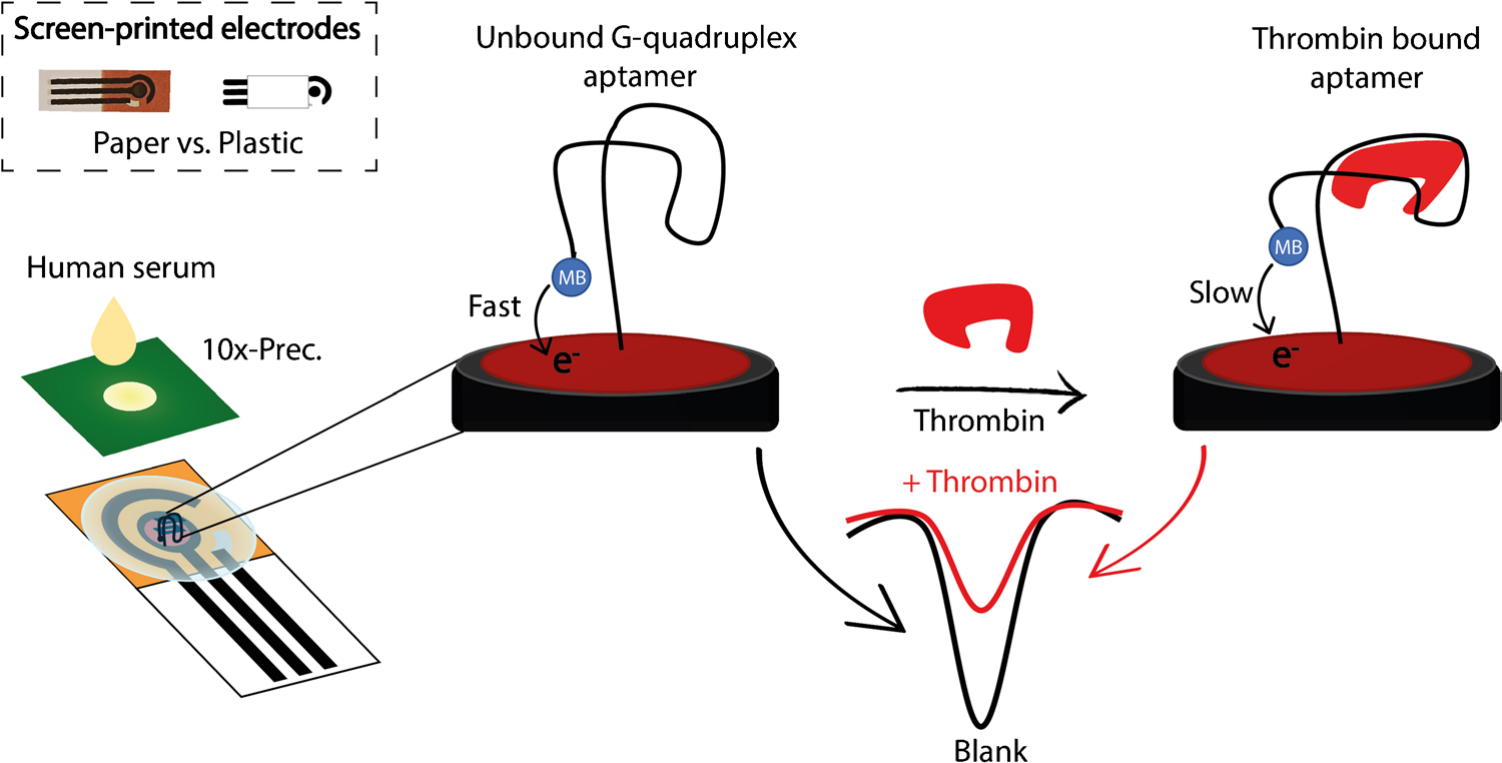
Schematic representation of the electrochemical device for the signal-off determination of thrombin at the gold nanoparticle-modified screen-printed electrode

In this work, we present the development of an electrochemical aptasensor for thrombin detection that combines sustainability with high analytical performance. To validate its performance, the aptasensor was applied towards spiked human serum, comparing the performance of the gold standard plastic-based and porous chromatographic paper as the manufacturing substrates. As reported in Fig. 1, both the plastic-based and paper-based screen-printed electrodes have been designed and analytically characterized.

## Experimental section

### Materials and apparatus

Chloroauric acid (HAuCl_4_), sodium borohydride, sodium citrate, sodium chloride (NaCl), PBS tablets (140 mM NaCl, 10 mM phosphate buffer, 3 mM KCl), tris(2-carboxyethyl) phosphine (TCEP; C_9_H15O_6_P), 6-mercapto-1-hexanol (MCH, C_6_H_14_OS), human serum, and the thrombin human plasma target were purchased from Sigma-Aldrich (St. Louis, MO, USA). The thrombin-binding aptamer (TBA) probes were designed starting from the original and well-known sequence (5′-GGTTGGTGTGGTTGG-3′), including the extended variants, i.e., TBA-4 (5′-(CH_2_)_4_-GGTTGGTGTGGTTGG-3′), TBA-6 (5′-(CH_2_)_6_-GGTTGGTGTGGTTGG-3′), and TBA-8 (5′-(CH_2_)_8_-GGTTGGTGTGGTTGG-3′), were purchased from Biosearch Technologies (Novato, CA, USA). All the electrochemical measurements were carried out using a portable potentiostat PalmSens 4 (PalmSens, Netherlands) equipped with a multi-8 reader and interfaced to a laptop using PSTrace5.9. All potentials reported are referred to the Ag/AgCl pseudo-reference of the screen-printed electrochemical strips.

To evaluate the sensing architectures, two types of screen-printed electrodes (SPEs) were utilized: one made from office paper and the other from polyester-based printed strips [31, 32]. These electrodes were produced using a manual printing method, as detailed in previous research.

### AuNPs synthesis

AuNPs were synthesized following a previously documented method [33]. Prior to commencing the synthesis, the glassware and magnetic rod were thoroughly cleaned with aqua regia, a mixture of HCl and HNO_3_ in a 3:1 v/v ratio, and then rinsed with distilled water. Next, a piranha solution was used, consisting of H_2_SO_4_ and H_2_O_2_ in a 7:3 v/v ratio, followed by a further rinse with distilled water. This cleaning cycle was repeated three times to ensure maximum purity. For the synthesis of AuNPs, the reaction was conducted at room temperature in a laboratory flask. Nine milliliters of distilled water was mixed with 1 mL of HAuCl_4_ at a concentration of 0.01 g/mL and 2 mL of sodium citrate at the same concentration. Subsequently, 0.5 mL of sodium borohydride at a concentration of 20 mM was added. The resulting solution was left to stir in the dark overnight. Finally, the resulting AuNPs dispersion was stored at 4°C.

### Electroanalytical measurement

The measurements have been carried out through the use of square wave voltammetry, using the following experimental parameters: E begins of 0.0 V, E ends of −0.5 V, E steps at 0.001 V, amplitude at 0.01 V, and frequency at 50 Hz. Measurements were conducted on eight distinct SPEs concurrently by inserting the strips into the 8-channel multiplexer linked to the portable potentiostat. All measurements were carried out using a drop volume of 100 μL, and all currents were sampled after 30 min of the target addition. For all measurements, the signal change% was evaluated as follows: signal change%= (I_0_-I_target_)/I_0_%, where *I*_0_ is the signal obtained in the absence of thrombin and *I*_target_ is the signal obtained in the presence of thrombin. The same procedure was performed both for standard and human serum measurements.

## Results and discussion

The working electrode was first modified with a layer of gold nanoparticles and then covalently bound to a thrombin-binding aptamer (TBA) oligonucleotide conjugated to a redox mediator, methylene blue (MB). As shown, the presence of thrombin leads to a decrease in the current associated with the electron transfer of MB at the electrode due to a slower redox process. In the absence of thrombin, the aptamer is believed to remain in a relatively unfolded state. In this configuration, MB molecules attached to the aptamer can collide or bind to the electrode, facilitating electron transfer and producing a measurable redox signal. However, upon binding to thrombin, the aptamer undergoes a conformational change that stabilizes the formation of a G-quadruplex structure. This transition significantly alters the electron transfer pathway. In the G-quadruplex conformation, the MB molecules are positioned farther away from the electrode, which increases the electron tunnelling distance, thereby reducing the efficiency of electron transfer and decreasing the redox signal. This conformational change effectively inhibits the reduction of MB at the electrode, resulting in reduced signal. The key point is that the aptamer exists in a conformational equilibrium between its unfolded state and the folded state of the G-quadruplex. Thrombin selectively binds to the conformation of the G-quadruplex and stabilizes it, pushing the equilibrium toward the folded state and resulting in the observed signal change. Aptamer folding in the absence of thrombin allows for more efficient electron transfer, while G-quadruplex formation in the presence of thrombin reduces the signal [34, 35]. The whole system is described as a signal-off because the increase in the target level decreases in the voltammetric peak. In particular, it should be noted that the thrombin is specifically recognized by the aptamer through the formation of a G-quadruplex structure. To evaluate the detection of thrombin at both plastic-based and paper-based electrodes, different thrombin-binding aptamer (TBA) probes were designed starting from the original and well-known sequence (5′-GGTTGGTGTGGTTGG-3′), including the extended variants, i.e. TBA-4 (5′-(CH_2_)_4_-GGTTGGTGTGGTTGG-3′), TBA-6 (5′-(CH_2_)_6_-GGTTGGTGTGGTTGG-3′), and TBA-8 (5′-(CH_2_)_8_-GGTTGGTGTGGTTGG-3′).

All probes used in the development of the aptasensor were designed with a thiol group at the 5′-end and MB at the 3′-end, respectively, to allow the covalent attachment of probes on the gold nanoparticles and to electrochemical transduce the aptamer-thrombin binding [36]. The thiol group forms a strong interaction with the gold surface, creating a robust Au-S covalent bond that ensures stable attachment, resistant to environmental factors such as changes in pH, temperature fluctuations, and exposure to organic solvents. This significantly reduces the risk of aptamer detachment, even in complex biological matrices like human serum, where non-specific interactions are more likely to occur. Furthermore, covalent attachment minimizes non-specific interactions with other molecules in the sample, enhancing the sensor’s specificity and reducing background noise.

**Fig. 2:**
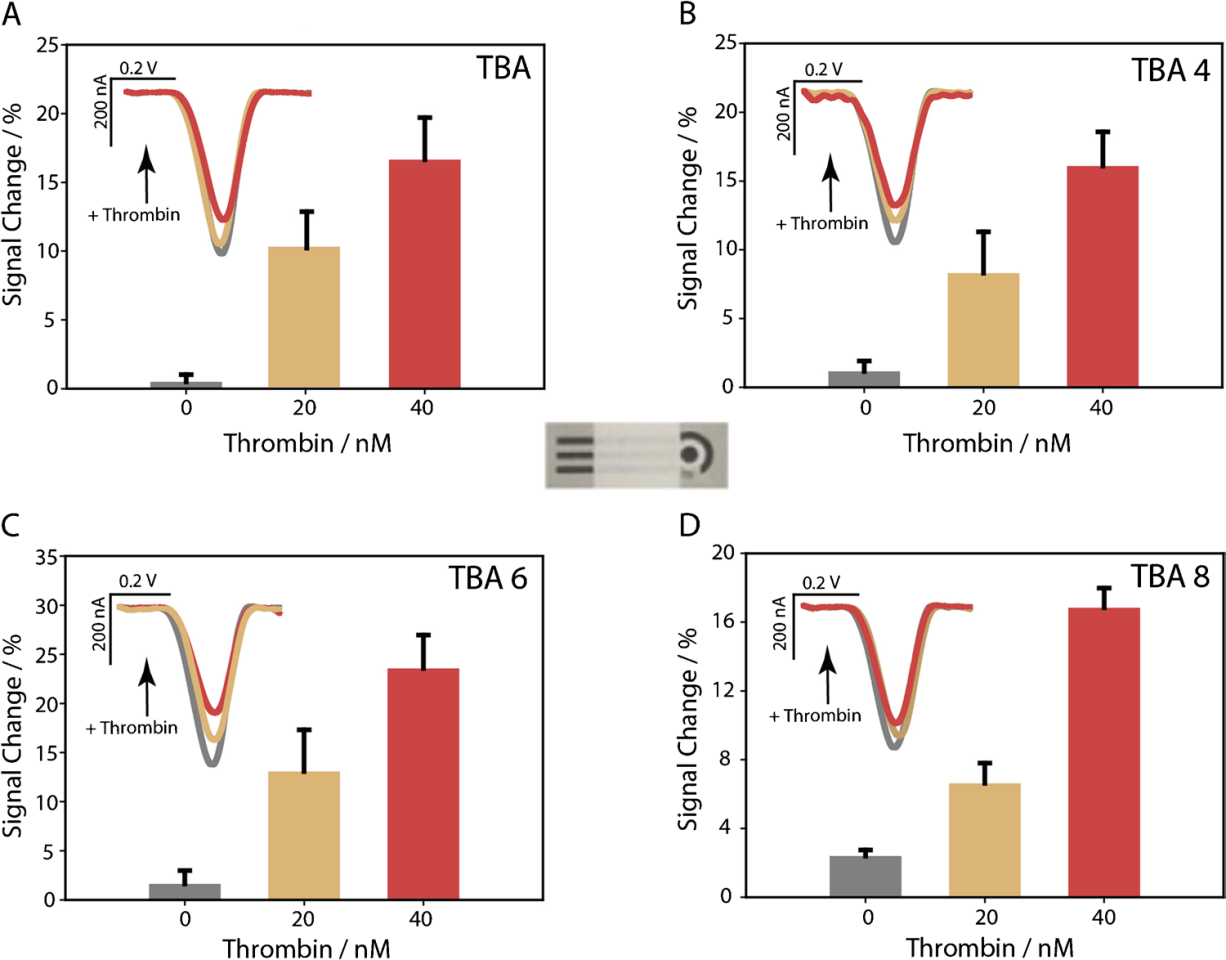
Signal change in the presence of 20 and 40 nM thrombin at the plastic-based screen-printed electrodes modified with TBA **A** in the absence of spacers and the presence of **B** 4, **C** 6, and **D** 8 carbon atoms spacers. All measurements have been carried out using square wave voltammetry and triplicates

As shown in Fig. 2, the plastic-based substrate (i.e., polyester) provided a maximum signal change of approximately 20% at the highest thrombin concentration tested (40 nM).

**Fig. 3:**
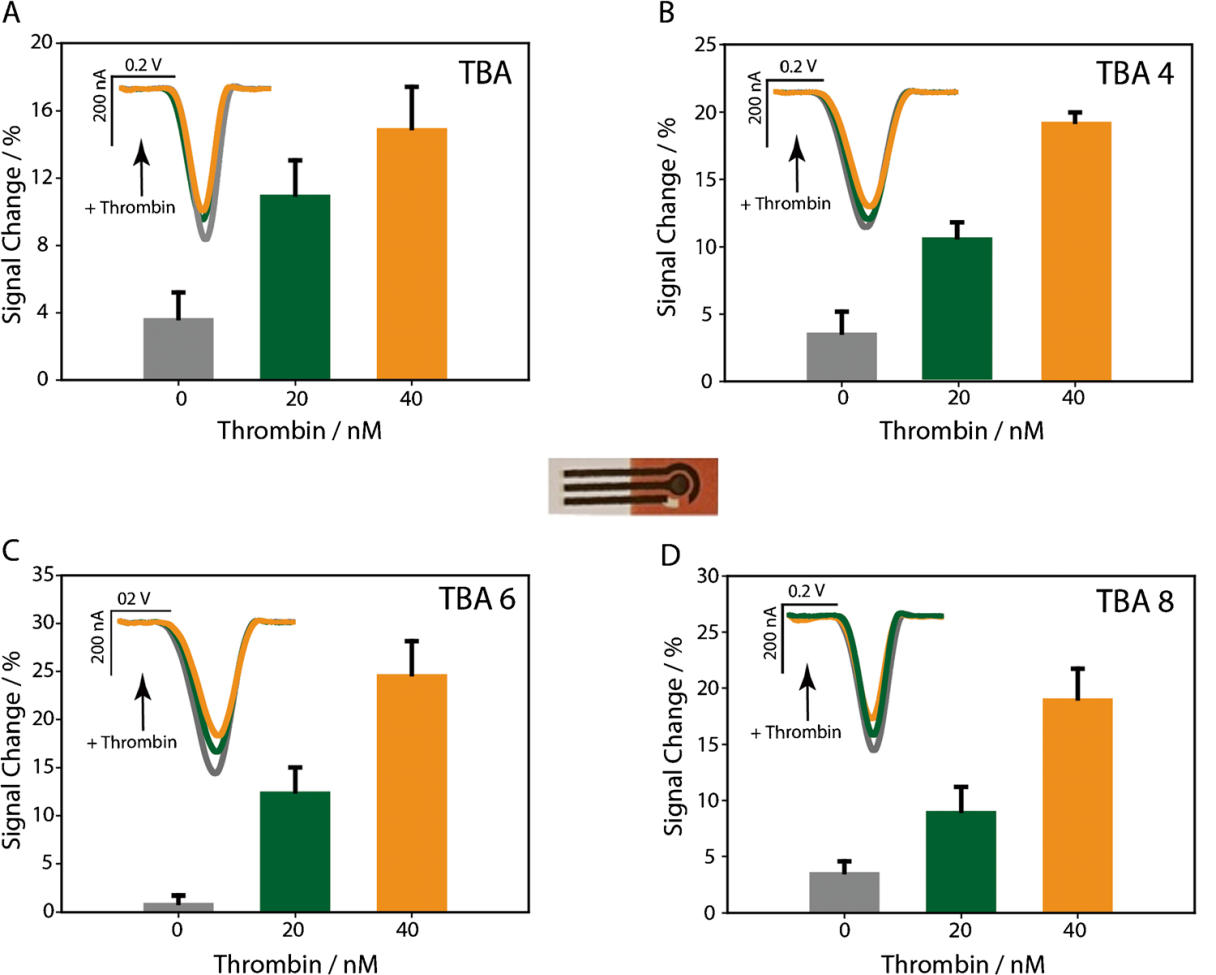
Signal change in the presence of 20 and 40 nM thrombin at the office paper-based screen-printed electrodes modified with TBA **A** in the absence of spacers and the presence of **B** 4, **C** 6, and **D** 8 carbon atoms spacers. All measurements have been carried out using square wave voltammetry and triplicates

Considering the highest concentration of thrombin, the TB-6 probe produced the maximum signal change, ~ 25%, while both the shortest and longest TBA-based probes resulted in lower signal changes, approximately 15%. A similar trend was also observed by exploiting office paper-based electrodes as the electrochemical platform, as observed in Fig. 3.

The signal changes for office paper-based substrates were roughly similar to those observed for the plastic-based substrates, within the experimental errors, thus confirming the effectiveness of the sustainable substrate.

The design of TBA-based aptamers with variable length spacers aimed to study how the length of the spacer affected thrombin recognition and the subsequent interaction between the MB and the electrode surface. The signal variation depends strongly on the ability of the spacer to modulate aptamer conformation and interaction with thrombin. Probes without a spacer, such as TBA-0, showed the weakest signal response due to structural rigidity, which limits the flexibility of the aptamer and hinders the formation of the G-quadruplex structure essential for effective thrombin binding. This rigidity also hindered optimal MB positioning for efficient electron transfer. In contrast, TBA-6, with its 6-carbon spacer, demonstrated superior performance by achieving a balance between structural flexibility and electron transfer efficiency. Shorter spacers, such as TBA-4, limited aptamer folding, while longer spacers, such as TBA-8, introduced steric hindrances that disrupted thrombin binding. These results underscore the importance of spacer length in improving thrombin recognition and electrochemical signal generation. In addition to maintaining proximity between the MB and the electrode, the spacer plays a crucial role in increasing the conformational flexibility of the aptamer. This flexibility allows the aptamer to switch more efficiently to its G-quadruplex structure upon thrombin binding, ensuring proper orientation and distance for optimal interaction with the MB. Consequently, this improves signal intensity. The 6-carbon spacer of TBA-6 proved to be the optimal configuration, which facilitates efficient binding to thrombin and maximizes signal variation. Among all substrates tested, TBA-6 consistently provided the most favorable conditions for evaluating probe-target interactions.

**Fig. 4:**
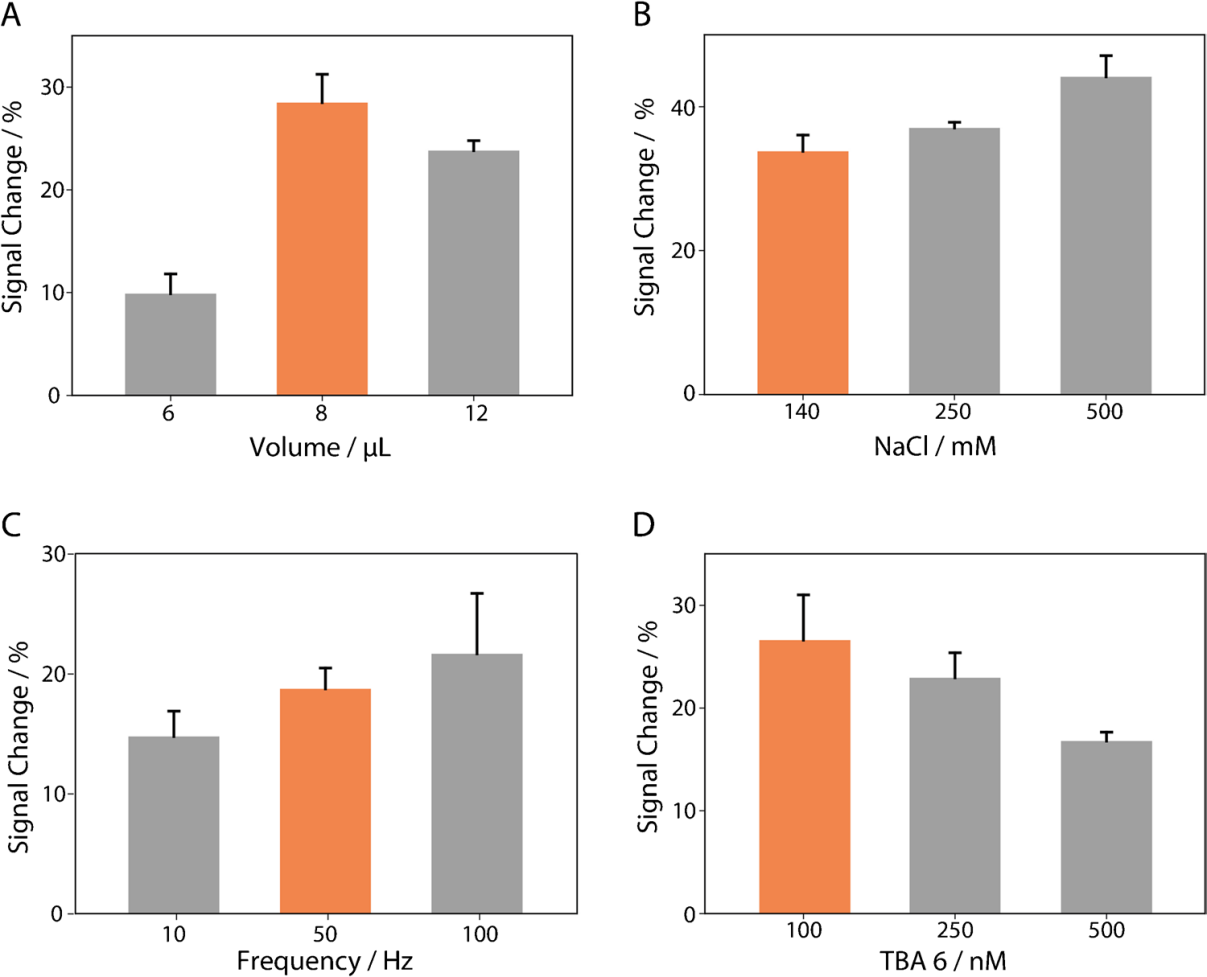
Optimizations of selected experimental parameters using square wave voltammetry as the electroanalytical technique. **A** Amount of gold nanoparticles (6, 8, and 12 μL), **B** sodium chloride concentration (140, 250, and 500 mM), **C** square wave frequency (10, 50, and 100 Hz), **D** TBA-6 probe used to modify the electrode (100, 250, and 500 nM). All histograms result from three replicates, performed with an electrode per measurement

After this preliminary evaluation, the TBA-6 aptamer has been chosen to continue with the development of the ultimate sensing platform, and experimental parameters such as the amount of gold nanoparticles, sodium chloride concentration, square wave frequency, and TBA-6 concentration have been considered to enhance the signal change variation in the presence of thrombin, as shown in Fig. 4.

Each measurement was thoroughly assessed for the percentage signal change resulting from the interaction between the probe and 40 nM thrombin. Optimization of gold nanoparticles played a key role in improving the overall performance of the sensor, particularly by enhancing the electrode surface coverage, facilitating better immobilization of the probe and increasing the electroactive area for electron transfer. The drop casting with 8 μL of gold nanoparticles, significantly improved sensor performance by enhancing key factors. First, the decision to use 8 μL of AuNPs was based on their impact on electrode surface coverage and electroactive area. This volume of AuNPs significantly improved sensor performance by increasing the electrode surface coverage, which provided more immobilization sites for aptamers. This allowed a higher density of aptamers, increasing the number of thrombin binding sites and improving the sensitivity of the sensor. In addition, the AuNPs expanded the electroactive area, which is critical for efficient reduction of methylene blue (MB) during the electrochemical reaction. A larger electroactive area allowed more MB molecules to interact with the electrode, producing a stronger and more stable electrochemical signal. These improvements in aptamer immobilization and electroactive area resulted in greater signal variation upon thrombin binding, thus improving the sensor’s ability to reliably detect thrombin. The choice of 8 μL represents a balance between maximizing sensor performance and minimizing material use, ensuring efficient thrombin detection without unnecessary nanoparticle consumption. Regarding sodium chloride, the observed trend was consistent with an improvement of the signal change with the increase in salt concentration. Even if the electrostatic repulsion between the target and the negatively charged probe decreases at higher ionic strengths, making the system more sensitive, a concentration of 140 mM of sodium chloride was selected because it was already present in the working phosphate buffer, thus eliminating the need for extra addition of salt, leading to a signal change of approximately 30%. Additionally, when performing square wave voltammetry, another critical parameter that required optimization was the frequency; it strongly affects the nature of signal change [37]. The square-wave frequency enables measurements while also allowing time for the diffusion of MB at the electrode, ensuring precise recording of electron transfer. As described in the literature, the signal change of this sensor class depends on the square-wave frequency, with the optimal value depending on the probe, redox mediator, and other specific characteristics of the device [38]. As a consequence of the study, 50 Hz resulted in a satisfactory compromise between sensitivity and repeatability, while 100 Hz resulted in too noisy measurements. The last parameter to be investigated was the concentration of aptamer used to modify the working electrode’s surface. Regarding the concentration of aptamers, we found that although a high concentration of aptamers may initially lead to higher electron transfer (and thus higher current), it does not necessarily improve sensor performance. At high concentrations, aptamers on the electrode surface can become overcrowded, causing steric hindrance. This reduces the ability of aptamers to bind effectively to thrombin, as aptamers are not well distributed and interfere with each other. As a result, despite the increase in the number of aptamers, their affinity for the target decreases, resulting in reduced sensitivity [39]. In contrast, an intermediate concentration of aptamers, such as 100 nM, provides the best balance. This concentration allows a well-distributed arrangement of aptamers on the electrode surface, optimizing thrombin binding without compromising the electrochemical signal. The result is an optimal combination of high sensitivity, repeatability, and cost-effectiveness for sensor fabrication. This allows optimization of thrombin binding without compromising electron transfer capability. This balance ensures high sensitivity, repeatability of measurements, and low cost for electrode modification. Even if not shown, a 50-nM concentration was also interrogated, but the recorded current was very low and unsuitable for further investigations.

**Fig. 5:**
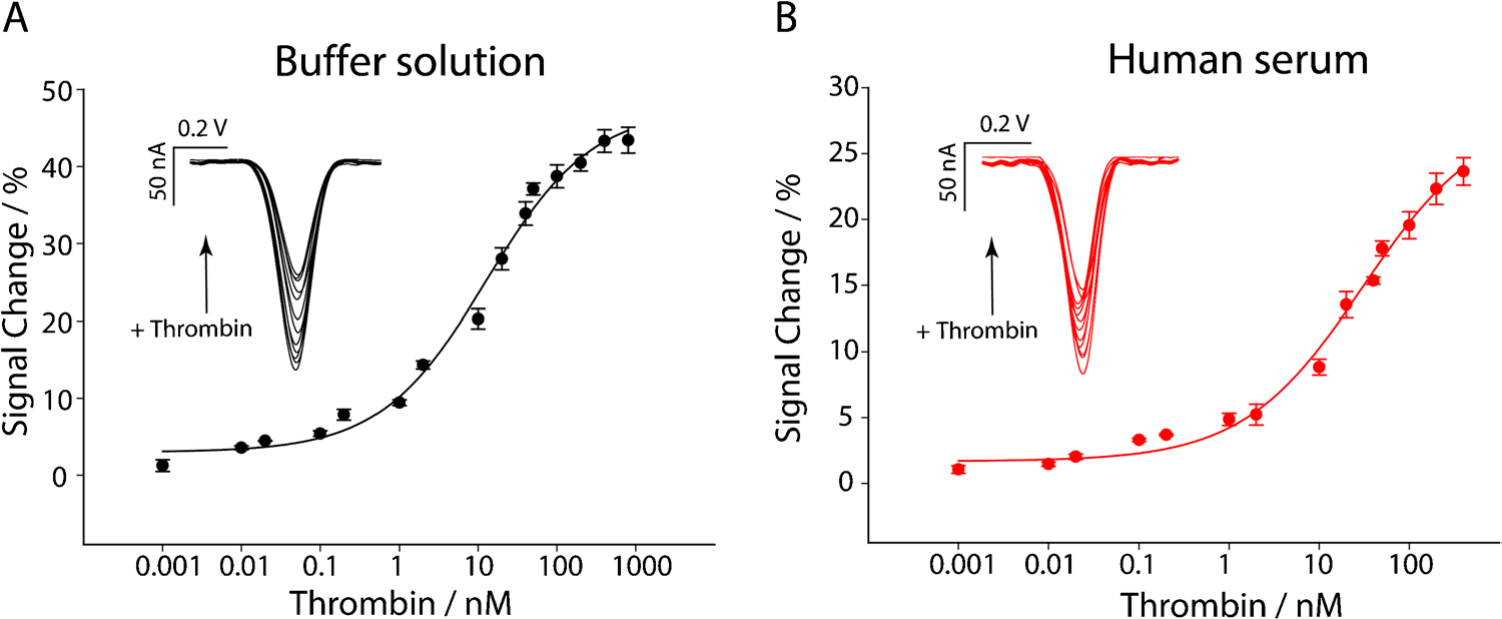
Calibration curves and square wave voltammograms obtained in **A** buffer solution by testing different concentrations of thrombin from 1 pM to 800 nM and **B** in human serum of thrombin from 1 pM to 400 nM. All the measurements have been carried out in triplicate. Experimental parameters: t equilibration=5 s, E start = 0.0 V, E end = −0.5 V, E step = 0.001 V, amplitude = 0.01 V, frequency = 50 Hz

Subsequently, the office paper-based platform was challenged in the presence of various concentrations of thrombin both in phosphate buffer and commercial human serum as the proof of concept. Accordingly, thrombin was spiked from in the range comprised between 1 pM to 400/800 nM, depending on the matrix, as reported in Fig. 5.

As reported in Fig. 5, a sigmoidal trend was observed for both solutions tested, as typical for aptasensors. This behavior is typical of affinity-based biosensors, where the interaction follows a Langmuir-type binding model. Initially, thrombin binds rapidly to the aptamers, followed by a slower increase as the thrombin concentration rises, leading to the sigmoidal curve. This response is indicative of cooperative binding, where multiple aptamers interact with thrombin molecules, resulting in a nonlinear increase in signal intensity. Additionally, the sigmoidal shape reflects the balance between aptamer surface density and available binding sites, which can become saturated at higher concentrations, causing the response to plateau [40, 41].

All points shown in the curves are the result of three measurements that have been carried out using three office paper-based electrodes to avoid the platforms’ memory effect and provide the end user with a disposable tool. For the two curves, the limit of detection (LOD) was calculated by determining the thrombin concentration that caused a 10% change in signal intensity from the baseline signal, a common approach in evaluating biosensor performance. The 10% threshold was chosen because it represents a statistically significant shift in signal that is still clearly distinguishable from noise or baseline level, indicating the sensor’s ability to detect low concentrations of the analyte (thrombin) with reliability. To determine this limit, a range of thrombin concentrations, from 1 pM to 400/800 nM, was tested, and the corresponding signal intensities were recorded. The concentration that produces a 10% change in signal from baseline was defined as the detection limit. The portable electroanalytical aptasensor was able to detect thrombin down to 60 pM and 100 pM in buffer solutions and human serum, respectively. In particular, what should be noted is the absence of sample pre-treatment, making the whole approach more suitable for decentralized applications. To rule out potential non-specific signal changes due to aptamer displacement, control experiments were conducted where the device was exposed to PBS solution and human serum without thrombin. In these control assays, no significant decrease in the signal was observed, confirming that the signal change was due to thrombin binding rather than aptamer displacement. If displacement had occurred, a drop in signal would have been expected, even in the absence of thrombin, which was not observed. Additionally, the absence of signal variation when exposed to human serum further supports the specificity of the biosensor, indicating that non-specific interactions were minimal (see Figures S1 and S2 in ESI).

**Fig. 6:**
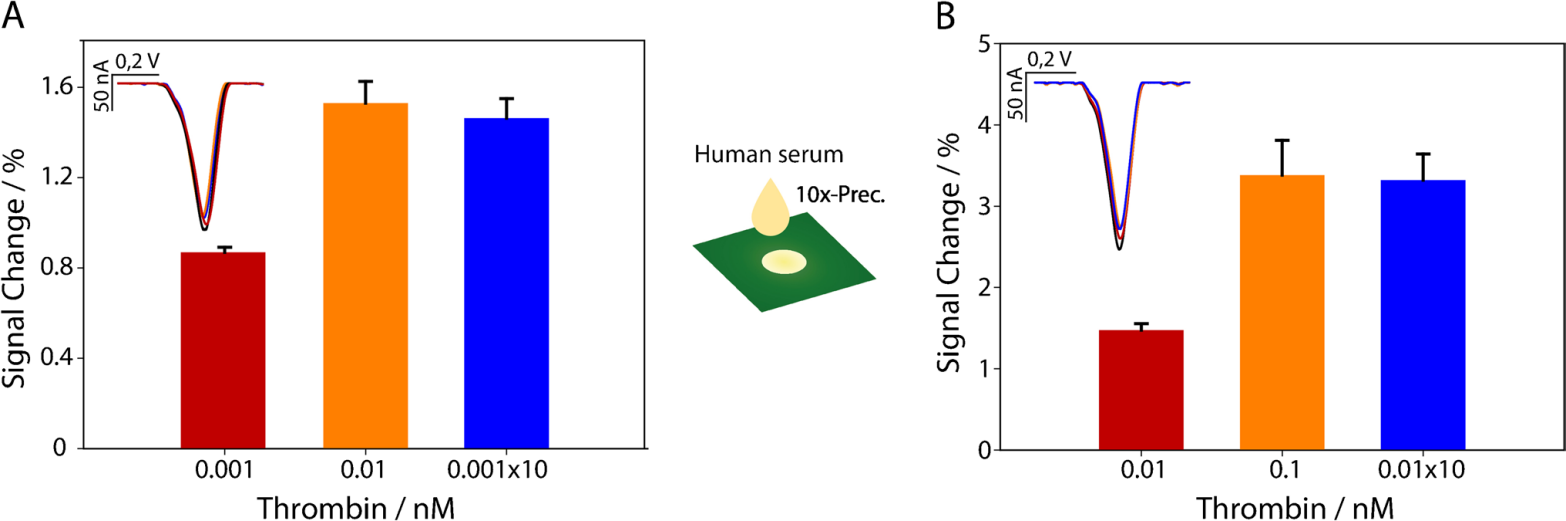
10× preconcentration of thrombin at paper-based chromatographic disc, using concentration equal to **A** 1pM and **B** 10 pM in human serum. All measurements have been carried out in triplicate

We performed a statistical validation of the method using a data set of 12 independent measurements at a thrombin concentration of 20 nM. The analysis yielded a mean signal variance of 20% with a standard deviation of 1.7, a relative standard deviation (RSD %) of 8%, a variance of 3, and a coefficient of variation of 0.08. These results confirm the robustness and reproducibility of the method, ensuring its reliability for analytical applications. Additionally, the efficacy of paper-based substrates was highlighted in lowering the effective detection limit. In fact, just by using a chromatographic paper-based disc (Whatman No. 1), it was possible to conduct a preconcentration of thrombin in human serum. Briefly, a paper-based chromatographic disc was used to store small aliquots of the sample to be quantified very simply. A 10× boosting was obtained by repeating drop casting of the sample on the paper-based disk 10 times. Each addition step of 5-μL sample was followed by the evaporation of water contained at room temperature, thus without employing an additional energy source and/or equipment. The 10× preconcentrated disc was released into a minimum amount of buffer solution and analyzed at the printed office paper-based electrode, as reported in Fig. 6.

As can be seen, after the 10× preconcentration simply operated by the adoption of porous paper, the effective thrombin level resulted equal to the nominal non-preconcentrated level, i.e., the signal change for the 10× preconcentrated 1 pM thrombin resulted equal to the signal change of the 10 pM solution. This was effective in improving of 10 times the detection limit of the whole approach, with the addition of only ~ 10 min due to the evaporation of water after each step of drop casting on top the paper-based disk. The developed biosensor demonstrated a limit of detection (LOD) in the picomolar range, comparable to that of laboratory-based tests, yet it offers a rapid and simple detection process, eliminating the need for complex sample preparation or sophisticated instrumentation. This capability underscores its significant potential for point-of-care diagnostics, where quick and accurate results are essential.

## Conclusion

This work represents a significant advancement in the development of a sustainable and portable aptasensor platform for thrombin detection. By utilizing paper-based substrates, we have designed an affordable, simple, and efficient sensor that is well-suited for clinical applications. The covalent immobilization of the aptamer on gold nanoparticles ensures the sensor’s stability and resistance to environmental factors. The inherent sustainability of office paper-based electrodes was further enhanced by the incorporation of a complementary porous paper-based disc, which effectively lowered the detection limit by approximately ten times, without the need for additional costs or complex equipment, making it comparable to laboratory-based tests. The biosensor achieved a limit of detection (LOD) in the picomolar range, which is crucial for clinical diagnostics, particularly in complex biological matrices like human serum. Moreover, the device demonstrated high specificity, as confirmed by control experiments showing no significant signal changes when exposed to human serum or PBS without thrombin. This ability to perform rapid, reliable, and accurate thrombin detection with minimal sample preparation offers promising opportunities for point-of-care diagnostics, particularly in the monitoring of thrombin-related diseases and conditions.

## Supplementary Information

Below is the link to the electronic supplementary material.

Supplementary file1 (DOCX 170 KB)

## Data Availability

The data supporting this article has been included as part of the Supplementary Material.
