# Supplementary material for: Cost-effective, user-friendly detection and preconcentration of thrombin on a sustainable paper-based electrochemical platform
Source: Anal Bioanal Chem. 2025 Feb 1;417(9):1863–72. doi: 10.1007/s00216-025-05764-9 (PMC11914299; doi:10.1007/s00216-025-05764-9)

**ESI**

**Cost-effective, User-Friendly Detection and Preconcentration of Thrombin on a Sustainable Paper-Based Electrochemical Platform**

**Ada Raucci^1^, Giuseppina Sorrentino^1^, Sima Singh^1^, Nicola Borbone^1^, Giorgia Oliviero^2^, Gennaro Piccialli^1^, Monica Terracciano^1*^ and Stefano Cinti ^1,3*^**

^1^Department of Pharmacy, University of Naples “Federico II”, Via Domenico Montesano 49, 80131 Naples, Italy.

^2^ Department of Molecular Medicine and Medical Biotechnologies, University of Naples Federico II, via Sergio Pansini 5, 80131 Naples, Italy. ^3^Sbarro Institute for Cancer Research and Molecular Medicine, Center for Biotechnology, College of Science and Technology, Temple University, Philadelphia, PA 19122, USA.

*Corresponding authors:

Email: monica.terracciano@unina.it; Orcid id: orcid.org/[0000-0001-6367-2419](https://orcid.org/0000-0001-6367-2419)

Email: [stefano.cinti@unina.it](mailto:stefano.cinti@unina.it); Orcid id: orcid.org/0000-0002-8274- 7452

**Production of the paper-based SPE**

Prior to screen-print the electrodes, filter and copy papers were patterned with wax through the use of a wax-printer (Xerox ColorQube 8580). In this work, three typologies of waxed configurations were adopted. The paper-based substrates were patterned with 1, 2, and 3 layers of wax. After a curing stage in the oven at 100 °C for 2 and 4 min, respectively for filter and copy paper, the wax penetrated the paper-based structures producing hydrophobic areas that contained the testing area. The electrodes were manually screen-printed using Ag/AgCl ink (Electrodag 477 SS, Acheson, Italy) for the reference electrode, and carbon ink (Electrodag 421, Acheson, Italy) for the working and counter electrodes.

**Engineerization of the paper-based SPE with the AuNPs/TFO hybrid**

A drop containing 8 μL of AuNPs was cast onto the working electrode, and after it dried the probe was immobilized following a protocol reported in literature. The first step is the reduction of 100 μM DNA in presence of 10 mM TCEP for 1 h. The resulting solution was then diluted to the chosen concentration (in the range of nanomolar) to be immobilized onto the AuNPs-SPE. A 20-μL drop of the probe was placed onto the working electrode area for 1 h at RT (in order to avoid solvent evaporation, the incubation was carried out in a humid chamber). SPE was gently rinsed with distilled water and incubated (in a humid chamber) with 2 mM C6-OH to passivate the empty spaces onto the working electrode. SPE was rinsed with distilled water and incubated in a humid chamber overnight at 4°C in the working buffer solution (50 mM phosphate buffer containing 150 mM NaCl (pH=7)). Prior to perform the measurements, each SPE was stabilized in working buffer until the current reached a constant value.

**Voltametric curves in buffer and serum solutions**


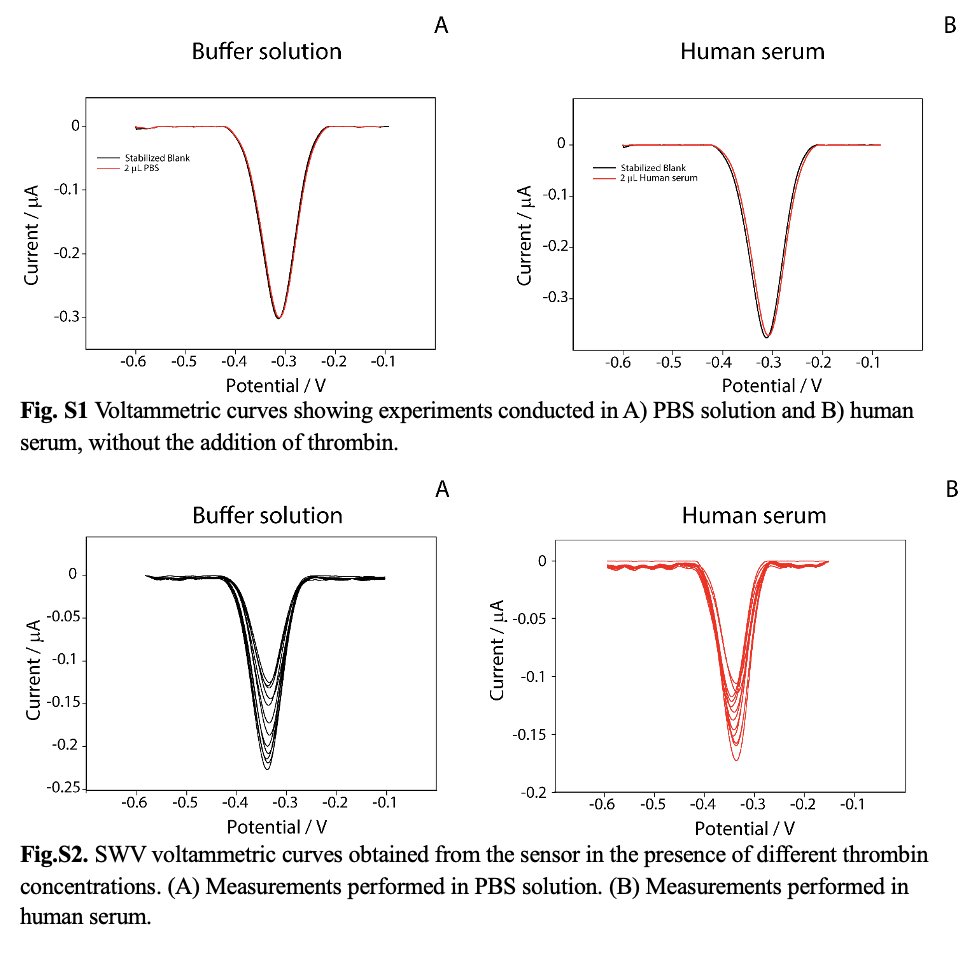

Supplement: Supplementary file 1 — Supplementary file1 (DOCX 170 KB) [file 216_2025_5764_MOESM1_ESM.docx]
